# Multimodal analysis of pretreated biomass species highlights generic markers of lignocellulose recalcitrance

**DOI:** 10.1186/s13068-018-1053-8

**Published:** 2018-02-27

**Authors:** Mickaël Herbaut, Aya Zoghlami, Anouck Habrant, Xavier Falourd, Loïc Foucat, Brigitte Chabbert, Gabriel Paës

**Affiliations:** 1 Fractionation of AgroResources and Environment (FARE) Laboratory, INRA, University of Reims Champagne-Ardenne, Reims, France; 2 Biopolymères Interactions Assemblages (BIA) Laboratory, INRA, Nantes, France

**Keywords:** Lignocellulose, Recalcitrance, Pretreatment, Saccharification, Correlation, Porosity, Lignin, Fluorescence

## Abstract

**Background:**

Biomass recalcitrance to enzymatic hydrolysis has been assigned to several structural and chemical factors. However, their relative importance remains challenging to evaluate. Three representative biomass species (wheat straw, poplar and miscanthus) were submitted to four standard pretreatments (dilute acid, hot water, ionic liquid and sodium chlorite) in order to generate a set of contrasted samples. A large array of techniques, including wet chemistry analysis, porosity measurements using NMR spectroscopy, electron and fluorescence microscopy, were used in order to determine possible generic factors of biomass recalcitrance.

**Results:**

The pretreatment conditions selected allowed obtaining samples displaying different susceptibility to enzymatic hydrolysis (from 3 up to 98% of the initial glucose content released after 96 h of saccharification). Generic correlation coefficients were calculated between the measured chemical and structural features and the final saccharification rates. Increases in porosity displayed overall strong positive correlations with saccharification efficiency, but different porosity ranges were concerned depending on the considered biomass. Lignin-related factors displayed highly negative coefficients for all biomasses. Lignin content, which is likely involved in the correlations observed for porosity, was less detrimental to enzymatic hydrolysis than lignin composition. Lignin influence was highlighted by the strong negative correlation with fluorescence intensity which mainly originates from monolignols in mature tissues.

**Conclusions:**

Our results provide a better understanding of the factors responsible for biomass recalcitrance that can reasonably be considered as generic. The correlations with specific porosity ranges are biomass species-dependent, meaning that enzymes cocktails with fitted enzyme size are likely to be needed to optimise saccharification depending on the biomass origin. Lignin composition, which probably influences its structure, is the most important parameter to overcome to enhance enzymes access to the polysaccharides. Accordingly, fluorescence intensity was found to be a rapid and simple method to assess recalcitrance after pretreatment.

**Electronic supplementary material:**

The online version of this article (10.1186/s13068-018-1053-8) contains supplementary material, which is available to authorised users.

## Background

Lignocellulosic biomass from plants is a promising alternative to fossil carbon resources as it is the most abundant renewable carbon resource in the biosphere, with an estimated annual production of 10–50 billion tons [1]. Dedicated crops such as poplar or miscanthus, and agricultural wastes such as wheat straw can be transformed to produce the entire range of compounds that are currently derived from petrochemistry without directly competing with the food industry via the valorisation of the plant cell walls’ components [2]. Cellulose and lignin are the main polymer of the plant cell walls and account for 50 and 30% of the terrestrial carbon in the biosphere, respectively [3, 4]. Cellulose fibrils are embedded in a matrix of covalently linked hemicelluloses and lignin that provide strength and mechanical support to the plant tissues [5]. The complex composition and the structural organisation of these biopolymers hinder their efficient valorisation through enzymatic hydrolysis, a phenomenon known as biomass recalcitrance [6–8]. Many factors are considered as critical to enzymatic hydrolysis of the plant cell walls, such as lignin and hemicelluloses content, cellulose crystallinity and pore size [9, 10].

Biomass porosity is considered as one of the main factors influencing enzymes access to the polysaccharides. It is generally admitted than pores with a diameter of 5–10 nm are too small for enzymes to diffuse efficiently [10]. Classical techniques to estimate plant cell wall porosity such as solute exclusion [11] and Simon’s Stain method [12] only give an indirect indication of specific molecules accessibility to plant cell wall components according to their size. Electron tomography can be used to directly assess pores size but requires drying the samples which might induce possible collapsing of the pores [13]. Low-field nuclear magnetic resonance (NMR) spectroscopy was recently used to study the interactions and retention of water molecules by the plant cell wall constituents in pretreated spruce [14, 15] and bagasse samples [16] showing positive correlation between water accessibility and biomass hydrolysis. The analysis of water molecules microenvironment can also be used to assess biomass porosity in conditions similar to that of saccharification.

Biomass porosity is partially attributed to its lignin content [17]. Although lignin is recognised as one of the main factors hindering biomass conversion, understanding the influence of its composition and structure is still challenging. Lignin structure impacts enzymes access to cellulose microfibrils [9]. It has been observed that lignin from grass species have a lesser impact on saccharification because to its higher syringyl (S) units content that results in linear lignin, whereas guaiacyl (G) units would induced the formation of branched lignin [18]. However, studies have shown that the S/G ratio, often used as an indicator of lignin composition, had no impact on the saccharification rate of different poplar variants [19, 20].

Despite the many studies carried out on biomass recalcitrance, it is still challenging to understand how the structural and chemical features hinder enzymatic hydrolysis [10, 18]. To address this issue, three representative biomass species, namely wheat straw and miscanthus which are typical grass species and poplar as a representative hardwood species, were submitted to four different pretreatments (dilute acid, hot water, ionic liquid and acid-chlorite pretreatments) selected for their contrasted effects. Dilute acid (DA) pretreatment is considered as one of the promising and leading pretreatment methods and allows hydrolysing and removing hemicelluloses from the plant cell walls, making cellulose more accessible while producing lower amounts of fermentation inhibitors than concentrated acid pretreatments [6, 21]. Hot water (HW) pretreatment affects all plant cell walls’ fractions that are partially hydrolysed in the reaction medium, while the minor amount of solubilised lignin can undergo condensation and redeposition onto the fibres’ surface [6]. The use of ionic liquids (ILs) offers a selective fractionation of the plant cell wall compared to other conventional pretreatments [21]. ILs are able to dissolve large amount of cellulose, but also lignin in relatively mild conditions [6], and they have also been used to dissolve the whole lignocellulosic complex [22, 23]. The IL 1-ethyl-3-methylimidazolium acetate has shown good abilities in dissolving big biomass particles compared to other imidazolium-based ILs [24]. The recovered biomass is more prone to enzymatic hydrolysis as cellulose is more porous and more essentially amorphous [21–23]. Acid-chlorite delignification (CHLO) has long been used in the paper industry to remove lignin from plant material with only trace solubilisation of the polysaccharides depending on the biomass species [25, 26]. CHLO delignification still is the most popular and established laboratory method for the removal of lignin from biomass [27].

These combinations of biomass species and pretreatments methods allowed generating a set of contrasted samples. A multimodal analysis was carried out using microscopy, wet chemistry and spectroscopy techniques to evaluate the changes in morphology, structure and chemical composition induced by the pretreatments. The data obtained were then scrutinised to determine the influence of biomass features on samples’ saccharification yield and to identify generic factors of biomass recalcitrance for predicting saccharification.

## Methods

### Plant materials

Wheat straw (*Triticum aestivum*) was grown in Champagne-Ardenne (France) and harvested at grain maturity stage. Internode 3 was isolated and cut in 2-cm-long fragments that were then halved in the longitudinal axis using a disposable blade. Miscanthus (*Miscanthus* × *giganteus*) was planted on experimental plots in the INRA site of Estrées-Mons (France) and late-harvested 2 years after planting. Basal stem region (Internode 4) was cut in fragments of 2 × 0.3 × 0.1 cm, and the rind was isolated from the pith using a razor blade. Poplar (*Populus nigra* × *deltoides*) was cultivated on experimental plots in Estrées-Mons (France) and harvested 2 years after planting. Fragments 2 × 0.6 × 0.2 cm in size were cut from the middle region of the main stem using a razor blade.

### Biomass pretreatments

#### Dilute acid and hot water pretreatments

Dilute acid (DA) and hot water (HW) pretreatments were performed on biomass fragments in mineralisation bombs equipped with Teflon cups (Parr) using a 2% (v/v) sulphuric acid solution or deionised water, respectively [28]. Pretreatments were carried out at a ratio of 1:30 (500 mg of biomass for 15 mL sulphuric acid or deionised water for the DA and HW pretreatment, respectively) using an oil bath to keep the fragments in the following conditions: 170 °C for 20 min for the DA pretreatment, 180 °C for 60 min for the HW pretreatment. Residence time and temperature were selected for each pretreatment in order to avoid plant cell wall disruption for microscopic analyses. The fragments were then cooled down in ice and thoroughly washed using deionised water and a 50% ethanol solution.

#### Ionic liquid pretreatment

Ionic liquid (IL) pretreatment of the biomass fragments was performed using 1-ethyl-3-methylimidazolium acetate with a biomass loading of 6% (m/v). Pretreatments were carried out using mineralisation bombs equipped with Teflon cups (Parr, USA). Samples were kept at 130 °C for 40 min in an oil bath. These conditions were selected to avoid miscanthus’ structure disruption for microscopic analyses. The fragments were then cooled down in ice, regenerated in deionised water at 4 °C, filtered using 20 volumes of deionised water and thoroughly washed in deionised water and 50% ethanol.

#### Sodium-chlorite pretreatment

Sodium-chlorite-acetic-acid delignification (CHLO) was performed based on the procedure described previously [26]. Pretreatments were carried out on 1 g of biomass fragments using 0.15 mL of acetic acid and 1.25 g of sodium chlorite added to 40 mL of water for 1 h at 70 °C. These 1-h incubations were repeated three times for wheat straw, six times for poplar and eight times for miscanthus samples in order to reach a homogeneous delignification rate based on fragments bleaching and chemical analyses. Fragments were then thoroughly washed using deionised water until water pH remains neutral.

### Enzymatic hydrolysis

Saccharification experiments were performed on the different biomass fragments using the Cellic CTec 2 ^®^ cocktail kindly provided by Novozymes A/S (Bagsværd, Denmark). Cellulase activity was measured (157.3 FPU/mL) by the filter paper method using Whatmann No. 1 filter paper as standard substrate. Enzymatic hydrolysis were performed in a 0.1 M citrate buffer at pH 4.8 containing 0.02% sodium azide with a biomass loading of 2% (w/v) and an enzyme loading of 90 FPU/g of biomass. The glucose released after 96 h of reaction was measured by anionic exchange chromatography. Samples of the reaction medium were filtered (PTFE, 0.45 µm) prior to injection on a 4 × 250 mm CarboPac PA-1 anion-exchange column (Dionex, Sunnyvale, USA) maintained at 22 °C. Elution was performed at a flow rate of 1.2 mL/min using the following gradient:

- MilliQ water at 100% for 40 min,
- Gradual decrease of the MilliQ water amount to 0% replaced by a 300 mM sodium acetate + 300 mM sodium hydroxide solution in MilliQ water during 15 min,
- 300 mM sodium hydroxide solution in MilliQ water at 100% for 10 min,
- Equilibration using MilliQ water for 5 min.

Detection was performed using a PAD-2 pulsed amperometry detector (Dionex), maintained at 30 °C and at basic pH using a 300 mM sodium hydroxide solution at a flow rate of 0.4 mL/min. Monosaccharides were quantified using both 2-deoxy-d-ribose as an internal standard and d-fucose, d-arabinose, l-rhamnose monohydrate, d-galactose, d-glucose, d-xylose, d-mannose, d-galacturonic acid monohydrate and d-glucuronic acid as standard solutions for calibration.

### Samples observations

#### Scanning electron microscopy (SEM)

Fragments’ overall morphology was investigated using an environmental tabletop electron scanning microscope Hitachi TM-1000 in low-vacuum mode without sputter coating. The transverse end surface of the samples was analysed using smaller fragments cut to a size of 0.5 cm placed on a graphite support with an inclination of 45°.

#### Field-emission scanning electron microscopy (FE-SEM)

60-µm-thin sections were coated with chromium in a sputter coater and examined with a JEOL 6700F field-emission scanning electron microscope using an accelerating voltage of 5 kV. High-magnification images were analysed using the ImageJ software (http://imagej.nih.gov/ij/) to determine the Feret diameter of the observed fibrillary structure. Ten fibrils were measured on two different images for each sample by manually applying a circular area covering the fibrils and values were averaged for comparison.

#### Confocal laser scanning microscopy (CLSM)

Morphology and fluorescence of cell walls were assessed using a fluorescence confocal microscope Leica TCS SP8 (Leica Microsystems, Germany) equipped with 63 × oil-immersion objective, a 405 nm laser line and Leica HyD hybrid detector. 60-µm-thin sections were infiltrated in 50% glycerol in 0.01 M phosphate buffer at pH 9 for 15 min prior to mounting in the same medium on a microscope slide as previously described [29]. Images were acquired 15 µm underneath the surface of the sections with a resolution of 2048 × 2048 pixels, a scan speed of 400 Hz, a numerical aperture of 1.4 and a 1× zoom factor. The laser power was set to 20% and fluorescence emission was detected using the HyD detector in counting mode. The Acousto-Optic Tunable Filter was set to select fluorescence emission from 415 nm to 650 nm during measurements. Images were treated using the Leica Application Suite X 2.0 (Leica Microsystems, Germany). Fluorescence intensity measurements were repeated five times for each sample in regions of interest with a diameter of 3 µm centred on the plant cell walls, and values were averaged for comparison.

### Nuclear magnetic resonance analyses

#### Low-field nuclear magnetic resonance relaxation measurements

Fragments’ porosity was assessed by low-field nuclear magnetic resonance (LF-NMR) relaxation measurements. 1-cm-long biomass fragments were soaked in water for 96 h prior to LF-NMR analysis using a Minispec mq20 spectrometer (Bruker) operating at 0.47 T (20 MHz proton resonance frequency) and equipped with a thermostated (± 0.1 °C) ^1^H probe. Fragments were packed in a 10-mm-diameter NMR tube to reach 1 cm height and left 10 min in the spectrometer for temperature stabilisation at 4 °C. The transverse (*T*_2_) relaxation curves were acquired using a Carr–Purcell–Meiboom–Gill (CPMG) sequence. The 180° pulse separation was 0.2 ms, 16,000 even echoes were collected and the 256 scans were acquired with a recycle delay of 7 s resulting in a total acquisition time of about 45 min. An inverse Laplace transformation (ILT) was applied to convert the relaxation signal into a continuous distribution of *T*_2_ relaxation components. For this purpose, a numerical optimisation method was used by including non-negativity constraints and L1 regularisation and by applying a convex optimisation solver primal–dual interior method for convex objectives (PDCO) [30, 31]. Similar NMR acquisition and data treatment protocols were implemented on controlled pore glass samples of known diameters (Sigma Aldrich; pore size: 8, 25, 50 and 100 nm). A linear relationship (*R*^2^ = 0.998) between *T*_2_ values and pore diameter was thus established and used to convert *T*_2_ distributions of biomass fragments into pore size distributions.

#### 2D HSQC NMR analysis

Lignin composition, as well as monomer linkages, was determined by 2D HSQC NMR analysis [32]. Milled samples (100 mg) were dissolved in 3.6 mL of DMSO and 1.8 mL of *N*-methylimidazole, and acetylated by addition of acetic acid for 4 h at room temperature. Samples were then precipitated in water, and centrifuged at 18,600*g* for 10 min (Beckman JLA-10.500 rotor). The pellets were washed twice and centrifuged again as described above. Acetylated samples (80 mg) were dissolved in 0.6 mL CDCl_3_ in a 5-mm NMR tube. NMR spectra were acquired and treated as described previously [33].

### Compositional analyses

Samples were milled to a granulometry of 80 µm before subsequent analysis.

#### Polysaccharides analysis

The sugar content was assessed by a two-step H_2_SO_4_ hydrolysis [28]. 125 µL of a 12 M H_2_SO_4_ solution were added to 10 mg of biomass samples for 2 h at room temperature under stirring, and then acid was diluted to 1 M for another 2-h incubation at 100 °C. Hydrolysed monomeric sugars were quantified by HPAEC-PAD as described previously.

#### Nitrogen content determination

Milled samples were oven-dried overnight at 80 °C, and 5 to 7 mg were weighted in tin capsule. Capsules were analysed using a EURO EA elemental analyser (Eurovector, Milan, Italy) equipped with a thermal conductivity detector. The samples were fully oxidised and nitrogen was converted into N_2_ and quantified using the Eager 200 software (Carlo Erba, Italy). Protein amount in the plant cell walls was then calculated by applying a nitrogen-to-protein conversion factor of ×6.25 [34].

#### Klason lignin content

The acid insoluble lignin content was quantified as described previously [33]. Milled samples (200 mg) were submitted to an acid hydrolysis by mixing with 2 mL of a 12 M H_2_SO_4_ solution for 2 h at room temperature. The acid solution was then diluted to a concentration of 2 M by addition of deionised water before an incubation of 3 h at 100 °C. The solid fraction was recovered, thoroughly washed and oven-dried at 105 °C to a constant weight. The Klason lignin content was calculated after correction for ash content which was determined by calcination at 550 °C for 4 h.

#### Thioacidolysis

The monomer composition of the labile ether-linked lignin fraction was determined by thioacidolysis as described by Lapierre et al. [35]. Milled samples (10 mg) were incubated in a solution of ethanethiol/BF3 etherate/dioxane (2.5/10/87.5v/v/v) with the addition of 1 mL of a 250 µg/mL tetracosane solution as an internal standard. Alkyl aryl ether linkage disruption reaction was carried out at 100 °C for 4 h, and then the reaction mixture was cooled down to room temperature and extracted three times using 25 mL of dichloromethane. Lignin subunits were then silylated before quantification using a gas chromatographer equipped with a fused silica capillary DB1 column (30 m  ×  0.3 mm) (J&W Scientific©) and flame ionisation detector. The temperature gradient was 160–280 °C at 2 °C/min [28].

### Data and statistical analysis

Wet chemical analysis experiments were carried out in triplicate, and the results are expressed as mean ± standard deviation. An analysis of variance (ANOVA) was performed on the obtained values followed by a Tukey test for comparison with the corresponding untreated samples with a significance level of probability set at *p* < 0.05. Statistical analyses were performed using the SigmaPlot 12.0 software (Systat Software, Chicago, USA).

## Results and discussion

### Morphological characterisation

The different pretreatment conditions were selected for the three biomass species regarding two important considerations: pretreatments had to be efficient enough to induce an increase in monosaccharides release during enzymatic saccharification while preserving structural integrity thereby allowing samples sectioning for photon and electron microscopic analyses. Focus was made on residence time and temperature of the different pretreatment processes, considered as the most impacting parameters [36, 37]. Weight losses induced by the different pretreatments are displayed in Table 1.

**Table 1:** Weight losses induced by the different pretreatments on each biomass species

Biomass	Pretreatment			
	DA	HW	IL	CHLO
Wheat straw	40.9 ± 1.4	35.5 ± 0.7	10.4 ± 1.2	20.3 ± 1.0
Miscanthus	26.8 ± 2.5	25.5 ± 0.9	1.5 ± 0.4	22.0 ± 3.0
Poplar	35.3 ± 3.0	31.4 ± 1.0	9.2 ± 1.3	22.9 ± 2.2

Weight losses are expressed in percentage of initial dry matter, as mean ± standard deviation

Overall, weight losses were more important for wheat straw and less important for miscanthus samples. The most important weight losses were observed after DA and HW pretreatments, while IL pretreatments induced the smallest matter removal, especially on miscanthus samples with only 1.5% loss. CHLO delignification removed similar amount of matter for each biomass species.

Pretreatment also modified the overall morphology of the samples, especially for the less rigid wheat straw fragments that appeared shrivelled, suggesting some important structural modifications (Additional file 1: Figure S1). The most notable difference was the change of colour of the pretreated samples, which is likely to be related to some modifications of lignin structure [38]. SEM images confirmed the structural alterations induced by pretreatments (Additional file 1: Figure S2). Pretreated wheat straw samples exhibited shrunk cell lumens compared to untreated samples, while some decohesion of outer structure of the samples could be observed for the other biomass species and especially for pretreated poplar.

### Microscopic characterisation

#### Fluorescence distribution within the plant cell walls

**Fig. 1:**
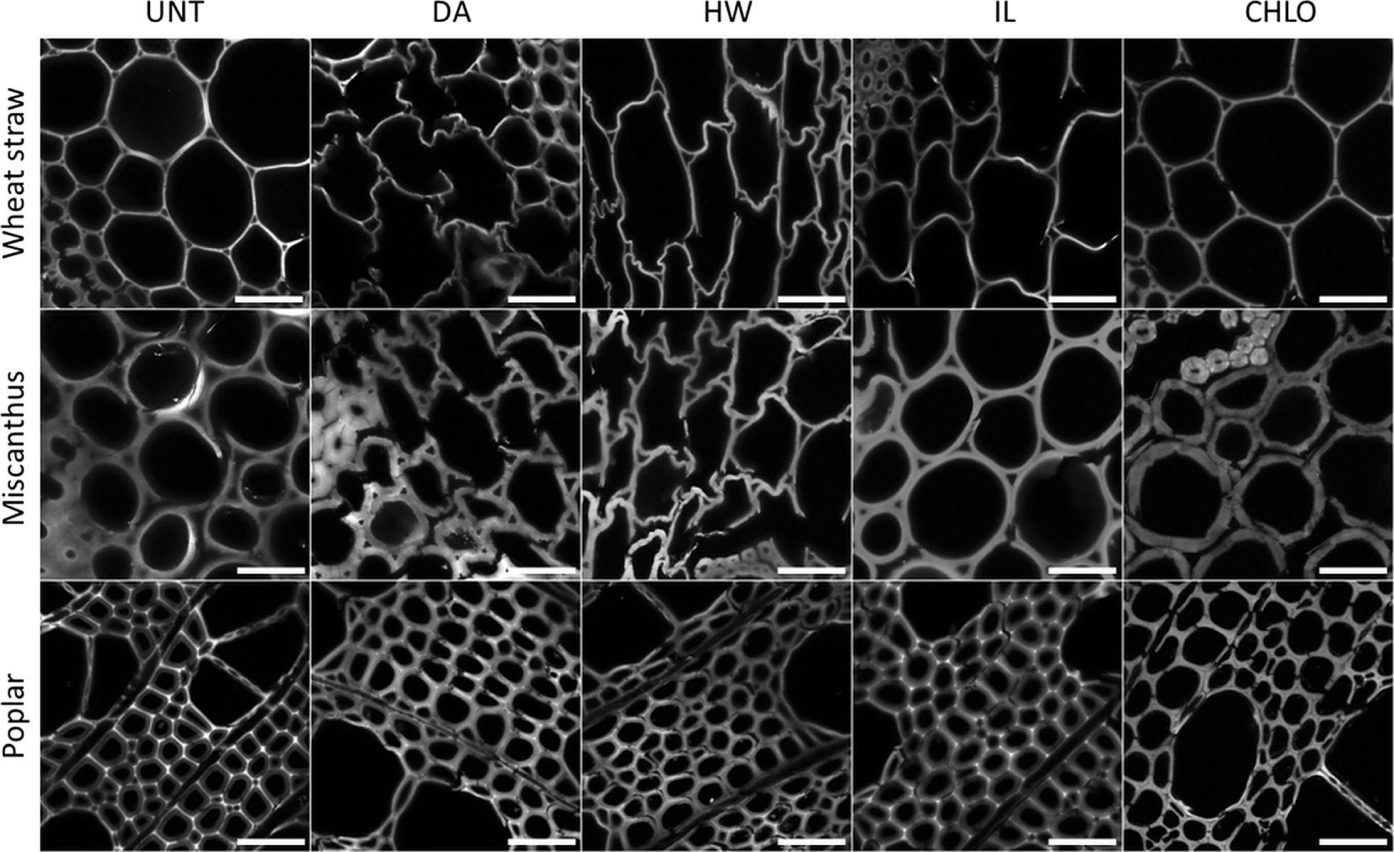
Confocal fluorescence images of untreated and pretreated biomass transverse sections before and after pretreatments. Images were taken in the interfascicular parenchyma region for wheat straw and miscanthus samples, and in the xylem region for poplar samples using the same laser and detector settings. Grey scale values were optimised for each image to overcome visibility issue due to differences in fluorescence intensity. Scale bar 50 µm

The structure of the plant cell walls was studied by imaging the autofluorescence of transverse sections using fluorescence confocal microscopy (Fig. 1). Autofluorescence in lignocellulosic biomass mainly originates from cell wall phenolic components such as lignin in mature plant cell walls, with very little or no contribution from the constitutive polysaccharides [39]. It can thus be assumed that autofluorescence gives an overview of lignin distribution within the plant cell walls.

Lignin distribution of both untreated wheat straw and miscanthus sections was uniform. After DA and HW pretreatments, the parenchyma cell walls of these biomass species were highly deformed, indicating some important structural changes (Fig. 1), while the more lignified sclerenchyma and vascular bundles were less impacted (data not shown). This observation could also be made for IL-pretreated wheat straw to a lesser extent, whereas IL-pretreated miscanthus retained its overall structure. After CHLO delignification, the cell walls of the miscanthus sections appeared disjoined, likely because of the removal of lignin from the middle lamella. The autofluorescence pattern of the poplar cell walls was modified after pretreatments. While light was emitted mainly from the cell corners and the middle lamella in untreated poplar section, the fluorescence distribution became more uniform after DA, HW and CHLO pretreatments, which could be due to important lignin redistribution inside the plant cell walls. Selig et al. proposed that lignin is fluidised during a pretreatment performed in neutral or acidic conditions above 120 °C, and is then able to move among the plant cell walls’ matrix to redeposit on its surface [40]. It is then likely that lignin underwent some condensation/repolymerisation reactions and redeposited more homogeneously in the plant cell wall. In contrast to previous pretreatments, the lignin distribution was retained after IL pretreatment that induces only weak removal of dry matter in our conditions.

**Fig. 2:**
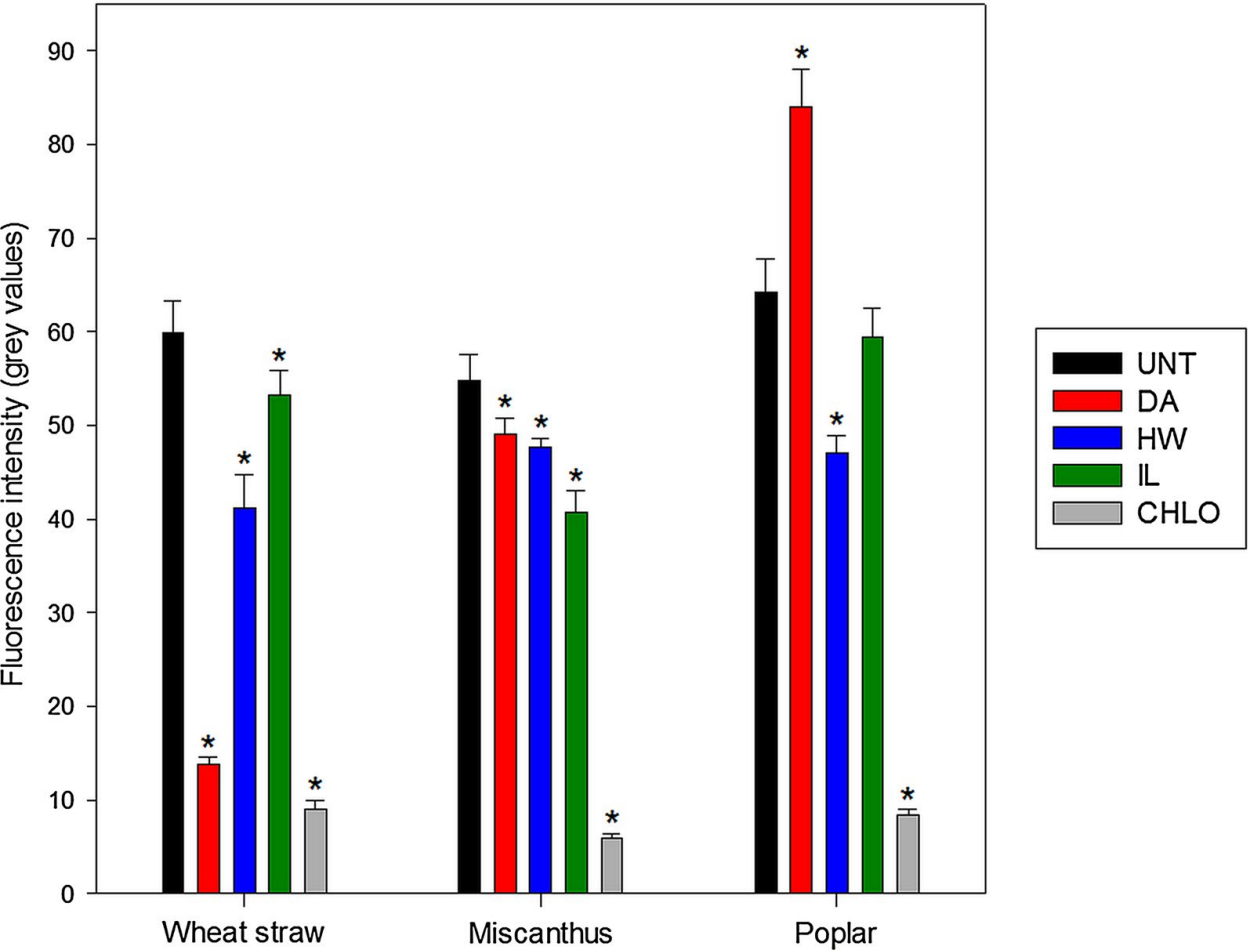
Cell wall fluorescence intensity of the untreated and pretreated biomass measured by fluorescence confocal microscopy. Measurements were performed in five different regions of interest, with the same laser and detection settings for all samples. Error bars indicate standard deviation. Asterisks indicate statistically significant difference between a pretreated sample and its corresponding untreated sample

The measured fluorescence intensities of the three untreated biomass samples were similar with mean values around 55–65 (Fig. 2). DA pretreatment had contrasted effect on the fluorescence intensity depending on the biomass species. It induced a huge decrease in light emission in wheat straw samples and a more moderate reduction in miscanthus samples, while fluorescence intensity increased in DA-pretreated poplar. Fluorescence intensity decreased significantly in all HW-pretreated samples. For IL pretreatment, a significant decrease was observed for wheat straw and miscanthus samples compared to untreated samples, but not for pretreated poplar samples (*p* = 0.261). Fluorescence intensity was greatly decreased after CHLO pretreatment for all biomass species. The evolution of fluorescence caused by the pretreatment processes seemed to depend both on the pretreatment performed and on the considered biomass species. However, a decrease in fluorescence intensity was observed for almost all pretreated samples, especially for the CHLO-pretreated samples.

#### FE-SEM observations of cell walls

**Fig. 3:**
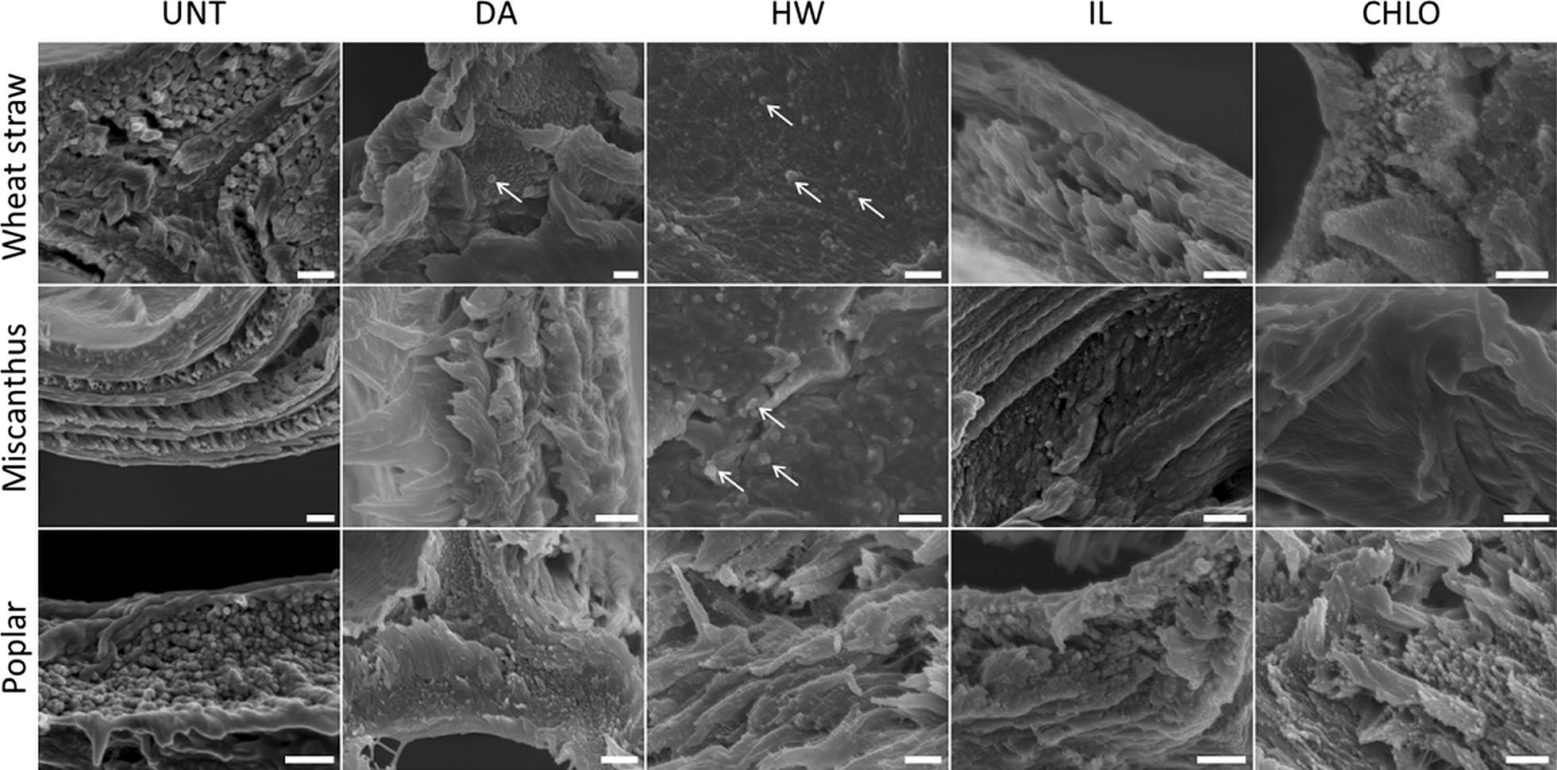
FE-SEM images of cell walls of untreated and pretreated biomass transverse section. Scale bar 500 nm

In order to gain more details on the structural changes of the samples, sections were observed using FE-SEM (Fig. 3).

Imaging transverse sections of the samples by FE-SEM allows observing ultrastructure of the cell wall that displays different layers with fibrillary structures likely attributed to cellulose. In DA-pretreated samples, cell wall layers remained visible in all sections, while observations were more complicated for HW-pretreated samples, especially for wheat straw and miscanthus. Nevertheless, some structures displaying larger diameters (100–200 nm) (Fig. 3, white arrows) were assumed to be lignin droplets [40]. Similar droplets have been observed after DA pretreatments of miscanthus fragments [41] and HW-pretreated wheat straw fragments [42]. CHLO pretreatment also greatly impacted layer structures, while IL pretreatment seemed to have a lower impact on cell walls’ overall morphology.

**Fig. 4:**
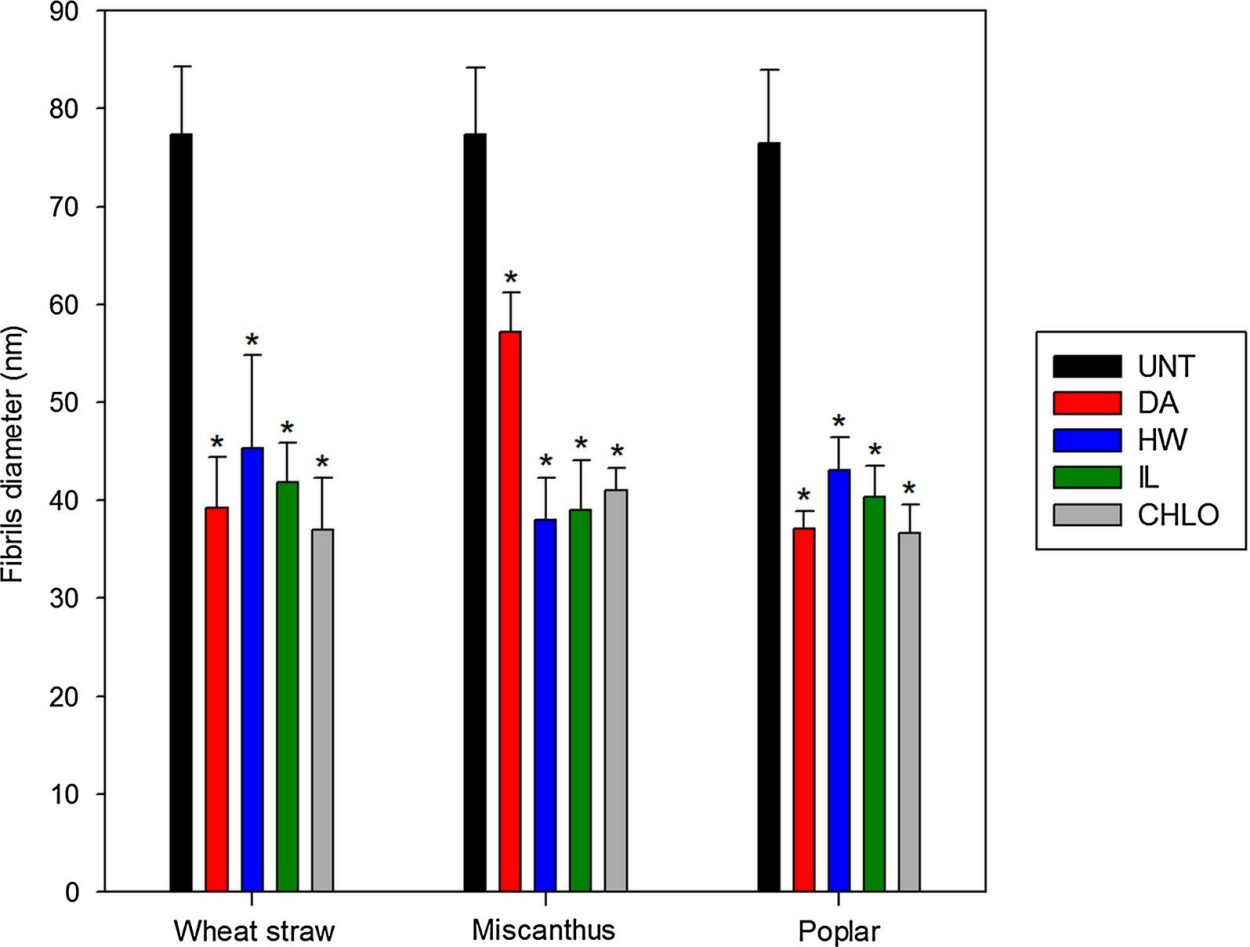
Macrofibrils diameter in untreated and pretreated biomass samples measured based on FE-SEM observations. Error bars indicate standard deviations. Asterisks indicate statistically significant difference between a pretreated sample and its corresponding untreated sample

Macrofibrils diameter was measured and averaged in order to assess the impact of the different pretreatments on plant cell walls’ organisation (Fig. 4).

The fibrillary structures displayed a similar diameter around 80 nm in untreated samples. Smaller structures (16 and 20 nm) have been observed in the secondary walls of poplar [43] and gincko [44], respectively, based on FE-SEM imaging. The observed fibrillary structures were then likely to be aggregates of macrofibrils. All pretreatments induced a reduction of the size of these aggregates which diameter ranged from 35 to 45 nm for almost all pretreated samples. Only DA-pretreated miscanthus samples displayed a lesser reduction with a diameter of 57 nm.

### Porosity

**Fig. 5:**
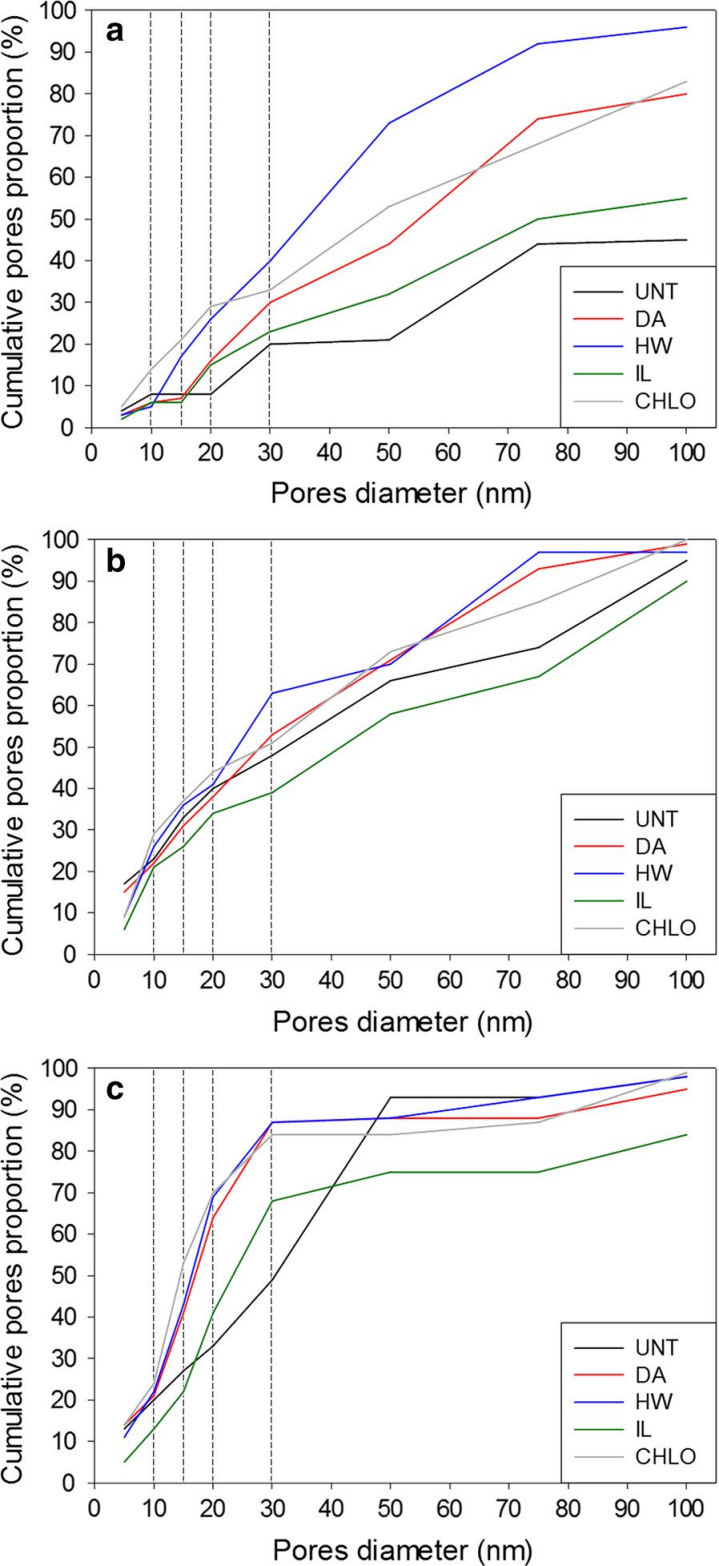
Cumulative pore size distribution of untreated and pretreated samples. Pore proportions are expressed as a cumulative percentage of the overall samples’ porosity. **a** Wheat straw, **b** miscanthus and **c** poplar

As the different microscopy techniques used to image the samples showed important modifications of the cell wall structure after pretreatments, change in porosity was also investigated. The proportions of pores in different porosity ranges were measured using NMR analysis of the relaxation time of water absorbed within the samples (Fig. 5). NMR was selected as it allowed working in conditions similar to those of enzymatic hydrolysis, in particular with hydrated samples.

Untreated samples displayed variations in their porosity pattern. While wheat straw showed a low amount of small pores with only 45% of the pores having a size below 100 nm, almost all pores were comprised below this threshold for untreated poplar and miscanthus samples. DA, HW and CHLO pretreatments all induced an increase in the proportion of pores with a size of 10–15 nm and more compared to the untreated samples. An increase in pores diameter had already been observed using NMR techniques in milled poplar after DA pretreatment [45]. The impact of IL pretreatment depended on the biomass species. Pretreated wheat straw samples exhibited a higher proportion of pores with a size comprised between 15 and 100 nm after pretreatments, while the porosity of miscanthus was decreased by the pretreatment process. IL-pretreated poplar porosity was also lowered, except for pores on the range 20–30 nm. An increase in the porosity above 10 nm is likely to allow a better penetration and diffusion of enzymes, considering the nominal diameter of 5.1 nm representative of the size of cellulases [46], but this factor must be interconnected with other structural and chemical parameters [10, 47].

### Chemical characterisation

#### Chemical composition

**Fig. 6:**
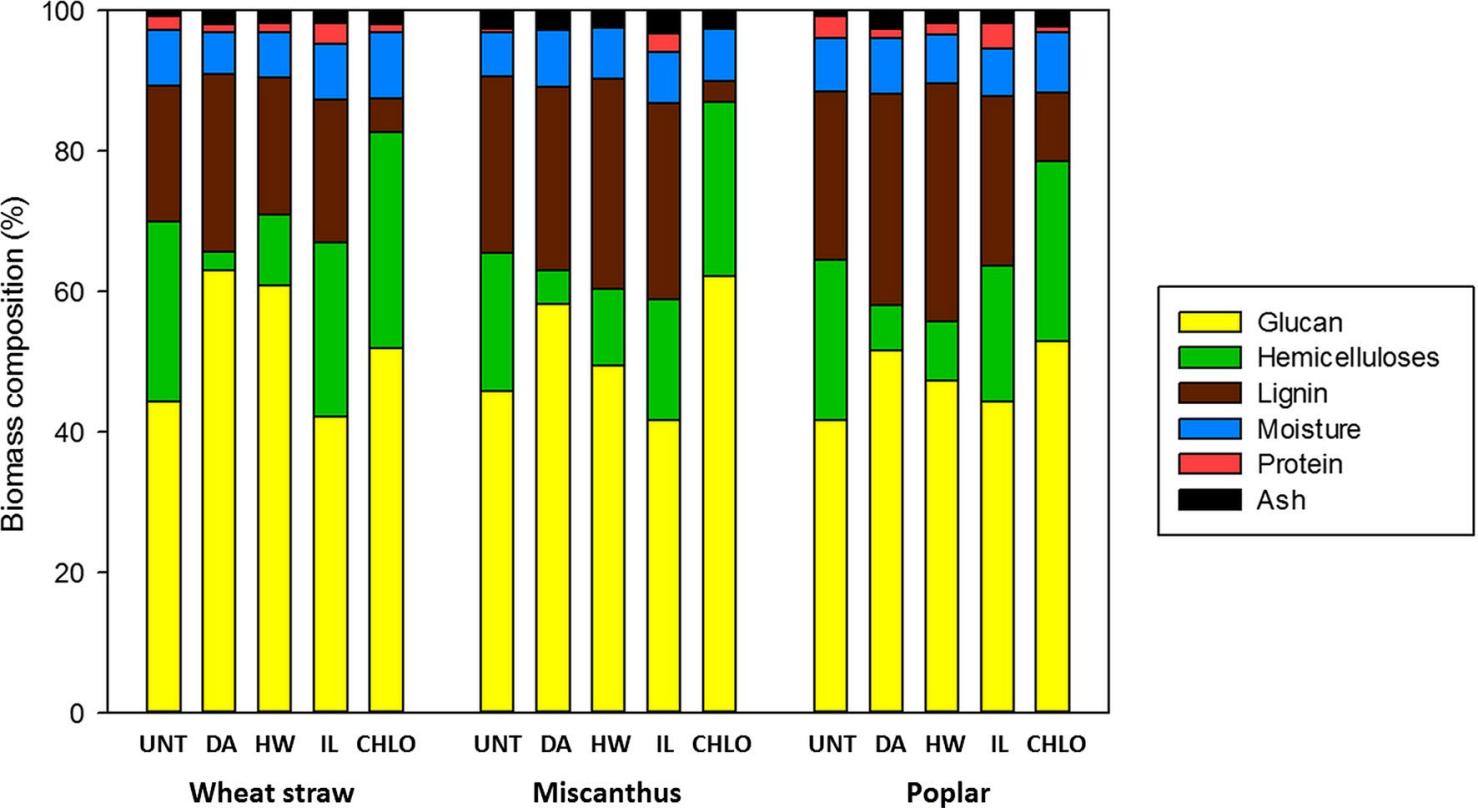
Chemical composition of the untreated and pretreated biomass samples

In order to better understand the structural changes induced by the different pretreatments, and especially the changes observed in lignin distribution, the chemical composition of the samples was investigated (Fig. 6).

Compositions are expressed as weight percentages (% w/w) of the dry biomass amount.

The glucan content of untreated samples was around 45% for both wheat straw and miscanthus samples, and 40% for poplar samples. DA pretreatment induced a decrease in hemicelluloses content for all biomass species, as expected [6], which was responsible for an increase in the relative glucan and lignin contents. Similar results were obtained with the HW-pretreated samples, but with a lower decrease in the hemicelluloses content. The glucan content was highly increased up to 61% in the HW-pretreated wheat straw samples, whereas the relative lignin content remained unchanged. For miscanthus and poplar HW-pretreated samples, the increase in glucan content was lower (from 46 to 49% and from 42 to 47%, respectively), while lignin content increased for both biomass species (from 25 to 29% and from 24 to 33%, respectively). It is noteworthy that miscanthus reacted in the same manner as poplar and displayed differences with wheat straw, whereas these two biomass species belong to the same family. The IL pretreatment slightly impacted the chemical composition of the three biomass species, with a small decrease of the hemicelluloses content, which is consistent with the small weight losses observed after pretreatment (Table 1). The fact that pretreatments are performed on fragments is likely responsible for the little removal of hemicelluloses and lignin as ILs are known to partially remove lignin and hemicelluloses, while almost all the initial cellulose can be recovered within the regenerated biomass [22]. CHLO pretreatment induced a decrease in lignin content for the three samples, resulting in an increase in the relative amount of glucose and hemicelluloses. Overall, the effects of the different pretreatments were similar regardless of the biomass species.

#### Lignin composition

**Fig. 7:**
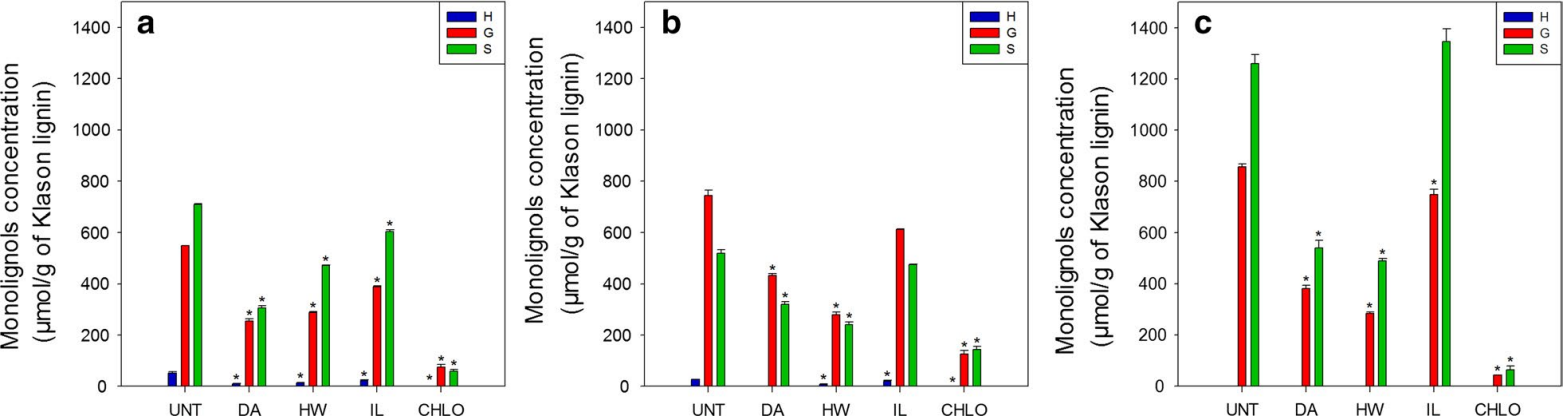
Monolignols quantification after thioacidolysis of untreated and pretreated biomass. **a** Wheat straw, **b** miscanthus, **c** poplar. Monolignols concentrations were assessed in triplicates. Error bars represent standard deviation. Asterisks indicate statistically significant difference between a pretreated sample and its corresponding untreated sample

Confocal microscopy images showed that lignin distribution in plant cell walls was highly affected by most of the pretreatments. Lignin was then analysed in more details using thioacidolysis and NMR. Thioacidolysis allowed the detection of the monolignols that are linked only through labile aryl ether linkages and that are more likely to be released during the pretreatments processes compared to condensed fractions [48], while NMR allows analysing the entire lignin polymer. The proportions of *p*-hydroxyphenyl (H), guaiacyl (G) and syringyl (S) units were measured after lignin thioacidolysis (Fig. 7).

Untreated poplar contained more ether labile G and S units and almost no detectable H unit as compared to wheat straw and miscanthus. Contrary to poplar and wheat straw samples, miscanthus released more G than S units. Similar labile monolignols proportions have already been observed after thioacidolysis of wheat straw [49], poplar [50] and miscanthus [28]. Different trends can be observed after each pretreatment, regardless of the biomass species. After DA and HW pretreatments, the amount of the three units decreased, whereas the lignin content of these pretreated samples was either unchanged or increased (Fig. 6). These pretreatments then probably induced a partial condensation of the remaining lignin. The monolignols contents of the IL-pretreated wheat straw slightly decreased probably because of a small condensation of lignin. No statistically significant differences were observed in miscanthus and poplar samples. For all CHLO-pretreated samples, thioacidolysis released very low amounts of monolignols as most of the lignin was removed from the plant cell walls (Fig. 6). Nevertheless, the decrease in monolignols in CHLO samples was more important than the reduction in lignin content, suggesting a condensation of the remaining lignin.

#### Lignin structural features

Complementary information was obtained on lignin structural features using 2D HSQC NMR allowing the characterisation of the whole lignin polymer. The proportions of labile β-O-4′ linkages which are the prevalent bonds in the lignin polymer were quantified as well as β-5′ phenylcoumaran and β-β′ resinol bonds which are considered as the most important condensed linkages strengthening lignin structure (Table 2) [51].

**Table 2:** Lignin inter-linkage proportions and syringyl-to-guaiacyl (S/G) ratio determined by 2D HSQC NMR spectroscopy

Biomass	Pretreatment	β-O-4′ linkages (per 100 aryl units)	β-5′ linkages (per 100 aryl units)	β-β′ linkages (per 100 aryl units)	S/G ratio
Wheat straw	UNT	46.10	4.46	3.81	0.67
	DA	14.85	ND	ND	0.64
	HW	5.94	8.29	ND	1.02
	IL	36.25	3.43	2.19	0.92
	CHLO	ND	ND	ND	ND
Miscanthus	UNT	41.64	6.32	2.91	0.46
	DA	37.06	ND	ND	0.53
	HW	13.77	9.79	ND	0.51
	IL	46.61	6.52	2.93	0.53
	CHLO	ND	ND	ND	ND
Poplar	UNT	54.66	5.92	6.06	1.08
	DA	39.66	5.99	10.76	0.56
	HW	34.98	5.96	8.37	1.46
	IL	59.99	4.75	7.38	1.22
	CHLO	ND	ND	ND	ND

Linkage proportions are expressed per 100 aryl units. *ND* not detectable

The proportion of β-O-4′ ether linkages was reduced after both DA and HW pretreatments for all of the three biomass species, with a stronger decrease induced by the HW pretreatment especially for wheat straw and miscanthus. This observation makes sense regarding the decrease in monolignol amount measured after thioacidolysis which disrupts only the β-O-4′ labile linkages [48]. After IL pretreatment, the proportion of β-O-4′ linkages was less modified in the wheat straw samples and was even increased for the poplar and miscanthus samples. This result suggests that in spite of the lack of variation in the lignin content, the polymer might have undergone some repolymerisation reactions instead of condensation as suggested by thioacidolysis data. Lower amount of condensed linkages was detected for all samples. In some cases, contour intensities did not allow quantification, especially for DA- and HW-pretreated wheat straw and miscanthus. However, these two biomasses exhibited similar modifications with an increase of the β-5′ linkages for the HW-pretreated samples, consistent with the previously described results and the observation of lignin droplets on this samples (Fig. 3). IL-pretreated wheat straw and miscanthus had a similar amount of both β-β′ and β-5′ bonds. All pretreatment induced similar modifications of the condensed linkages for poplar samples, with no effect on the β-5′ bonds and a slight increase in β-β′ bonds. For all CHLO-pretreated samples, contour intensities of the different peaks were too low to allow analysing the remaining lignin structure, as expected from the very low amount of lignin remaining in these samples (Fig. 6).

Important differences were also observed in syringyl-to-guaiacyl (S/G) ratios for the different biomass species. The S/G ratio of untreated miscanthus was lower than that of poplar and wheat straw untreated samples. Lignin with high S/G ratio is easier to remove because of the higher amount of ether labile linkages, which makes lignin more prone to deconstruction [52]. S/G ratio was increased after HW and IL pretreatments for wheat straw and poplar samples, indicating a degradation of the G units. Pretreated miscanthus samples showed no significant modification of their S/G ratio, suggesting a weaker impact of pretreatments on lignin.

### Enzymatic hydrolysis of the biomass samples

**Fig. 8:**
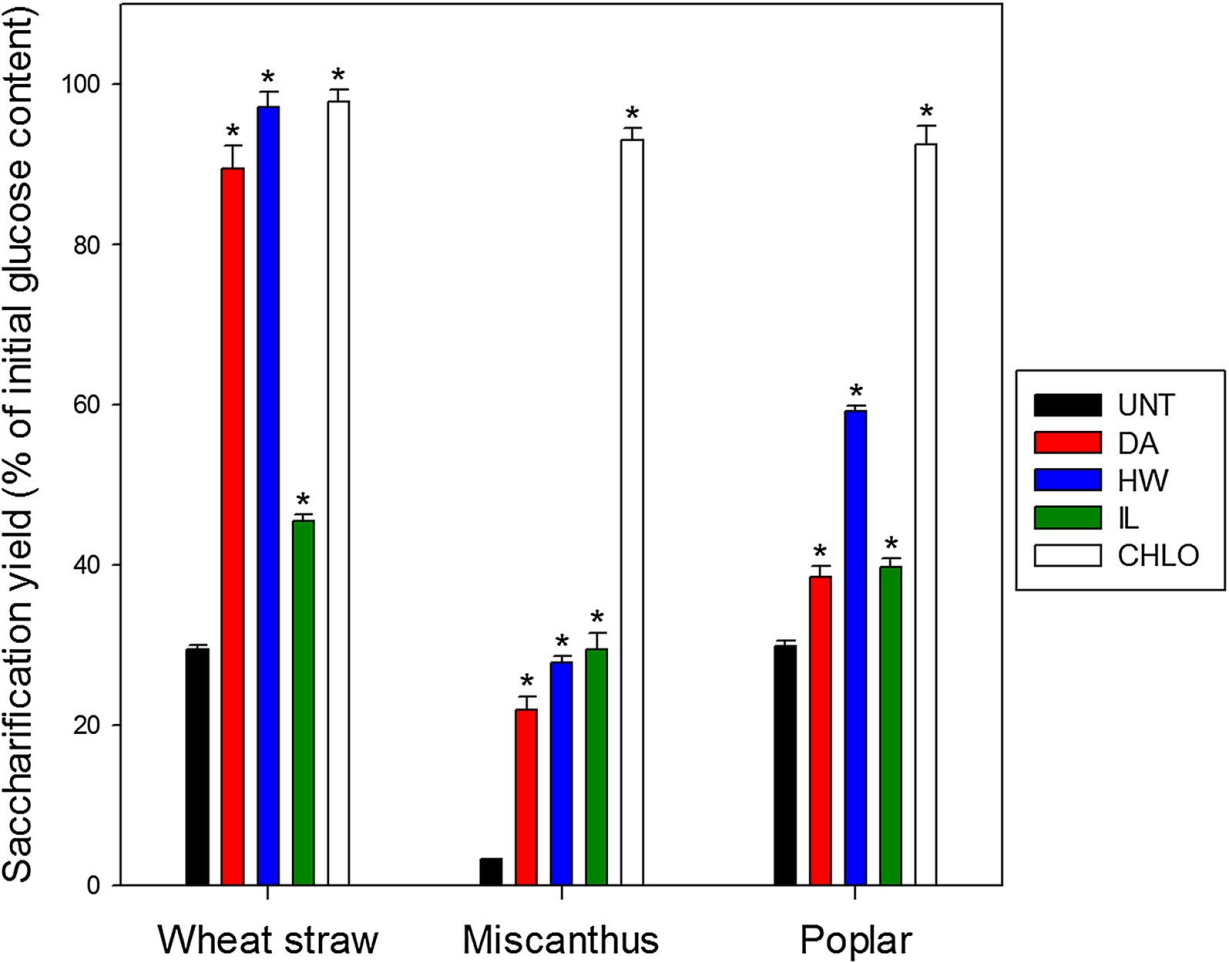
Saccharification yield after the 96-h enzymatic hydrolysis of untreated and pretreated biomass. Error bars indicate standard deviations. Asterisks indicate statistically significant difference between a pretreated sample and the corresponding untreated sample. *UNT* untreated; *DA* dilute-acid-pretreated; *HW* hot water-pretreated; *IL* ionic liquid-pretreated; *CHLO* sodium chlorite-pretreated

The effect of the different pretreatments on biomass digestibility was estimated by measuring the proportion of the initial glucose content that was released in the reaction medium during their enzymatic hydrolysis (Fig. 8). Saccharification experiments were performed with an enzyme loading of 90 FPU/g of dry matter. This value is much higher than standard conditions usually found in the literature (from 5 to 20 FPU/g of dry matter) [53–55] but is rationale based on the fact that substrates are fragments with much lower surface accessibility in comparison to milled samples.

Similar glucose yields (ca. 30%) were obtained from wheat straw and poplar samples, while untreated miscanthus was highly recalcitrant to enzymatic hydrolysis with a 10-time lesser glucose yield (ca. 3%). However, as the glucose content of all untreated samples was similar (Fig. 6), this lower saccharification yield is likely to result from the structure and the organisation of the plant cell walls’ components.

All pretreatments induced a statistically significant increase in glucose release after saccharification for each biomass. DA pretreatment lead to a more limited enhancement of the biomass digestibility than HW pretreatment for all samples, whereas the glucose content was more important in DA-pretreated samples (Fig. 6). Hemicelluloses removal then seemed to be an important but not essential parameter to increase saccharification yield. The increase in enzymatic hydrolysis efficiency induced by the IL pretreatment depended on the biomass species. This pretreatment was the least efficient on wheat straw samples (45%), whereas it was the second most efficient pretreatment on miscanthus samples (29%). The most efficient pretreatment was the CHLO delignification that allowed releasing more than 90% of the initial glucose content for all biomass species, with a fourfold higher glucose yield for wheat straw and poplar samples, and even a 40-fold higher glucose yield for miscanthus samples. DeMartini et al. also obtained higher glucose yield after CHLO delignification of poplar samples compared to other pretreatments [56].

The higher saccharification yields were obtained for the wheat straw samples, suggesting that wheat straw cell walls could be more easily modified and hydrolysed. This difference cannot be explained by the porosity of the cell walls as wheat straw samples exhibited the lowest amount of pores below 100 nm among the three biomass species (Fig. 5). Lignin could partially explain this better enhancement of saccharification. The lignin content of wheat straw samples was lower than that of the two other biomasses when comparing the pretreatments, with the exception of the CHLO pretreatment (Fig. 6). Moreover, all wheat straw samples displayed a lower amount of ether labile G units as quantified by thioacidolysis, and pretreated wheat straw samples also displayed lower amount of labile S units (Fig. 7). These results seem to indicate that a low amount of labile lignin is favourable to enzymatic hydrolysis. Altogether, the saccharification data seem to highlight the importance of lignin content and composition on the saccharification yield.

### Correlations between saccharification yields and biomass-related features

**Fig. 9:**
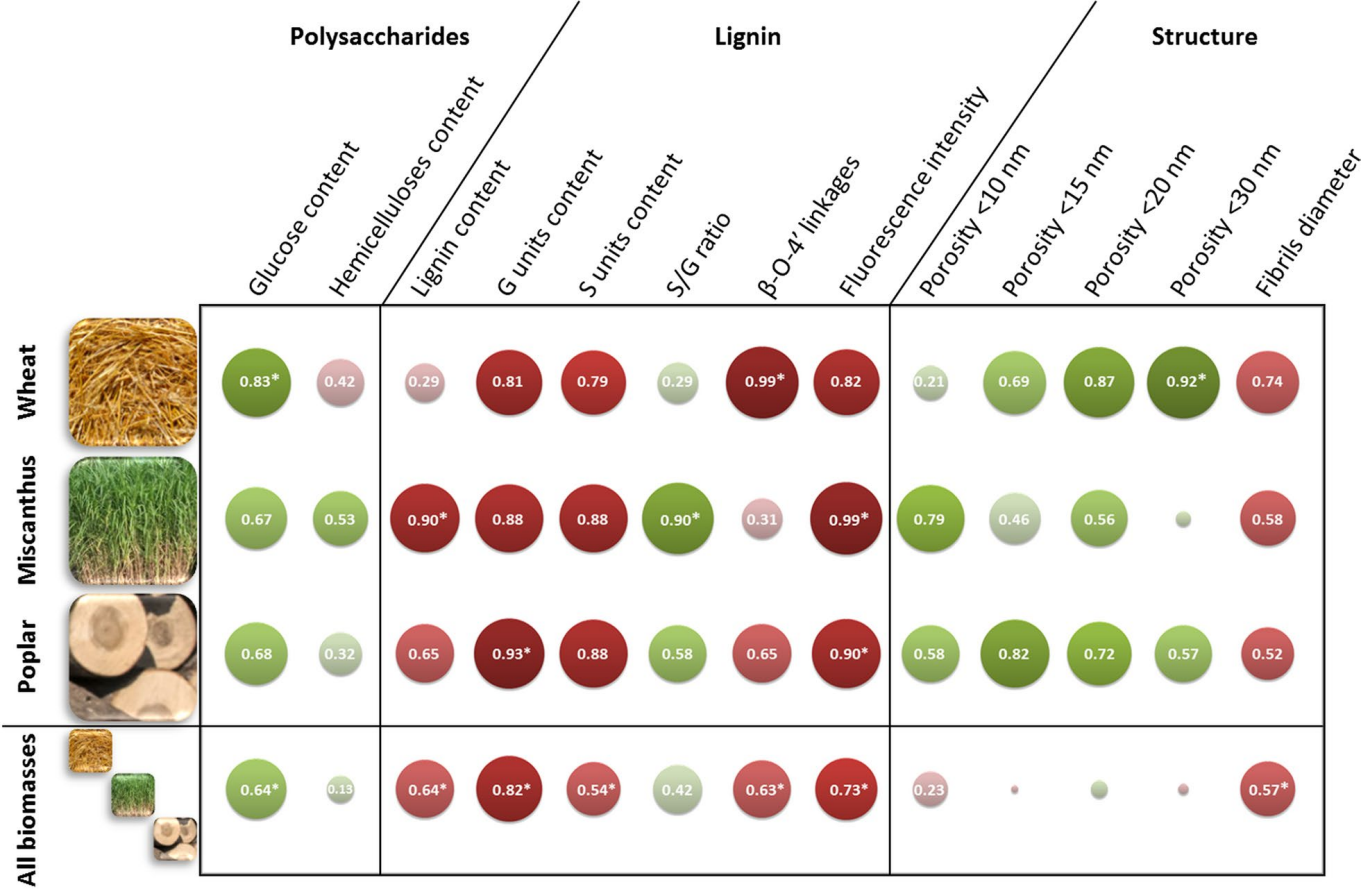
Pearson’s correlation coefficients between the saccharification yields after 96 h and biomass-related features. Discs diameter and colour shade display the strength of the correlations. Both positive and negative correlations are presented using the absolute value of the coefficients. Absolute values under 0.1 are not indicated on the corresponding disks. Positive and negative correlations are displayed in green and red, respectively. Asterisks indicate statistically significant coefficients with a *p* value below 0.05

Given the large spectrum of features characterised for our set of 15 contrasted samples, correlation coefficients were calculated to highlight the plant cell wall markers with a strong impact on biomass saccharification. One-to-one correlations were determined between the saccharification yield of the different samples (Fig. 8) and some of the measured characteristics of the biomass (Fig. 9). For lignin composition, only labile G and S units contents were considered as no H units could be detected after thioacidolysis of the poplar samples (Fig. 7). Regarding lignin linkages, only the β-O-4′ bonds were taken into account as the proportions of the condensed β-β′ and β-5′ bonds could not be determined for every samples (Table 2). For porosity measurements, only the proportions of pores below 10, 15, 20 and 30 nm were considered (dotted line in Fig. 5) since these pore sizes are in the same size order as the enzymes involved in hydrolysis.

#### Polysaccharide-related features

A positive correlation (0.64) was logically observed between the glucose content and the saccharification yield considering all samples. The highest coefficient between these two features was obtained for wheat straw samples (0.83), while miscanthus (0.67) and poplar (0.68) displayed lower and non-significant positive coefficients. However, the number of values was relatively low when considering each biomass separately (five different conditions), and *p* values were statistically significant only for very strong correlations compared to what is obtained considering all the samples. These values were consistent with the higher glucose yields obtained after hydrolysis of the wheat straw samples (Fig. 8). Considering all biomass species, no significant correlations were observed with hemicelluloses content (0.13) indicating that this parameter could not be related with saccharification efficiency, either in a positive or a negative way, whereas it was described as being detrimental to enzymatic hydrolysis in other studies [56]. Noteworthy, opposite effects of hemicellulose contents were observed for grass species like wheat straw and miscanthus. Some other features such as hemicelluloses organisation and interactions with both cellulose and lignin probably have more influence on recalcitrance than hemicellulose content alone.

#### Lignin-related features

Lignin-related factors displayed very strong negative correlation coefficients with saccharification efficiency either when considering the three biomass species together or separately. This was expected as lignin acts as a physical barrier that also unproductively binds enzymes [57, 58]. Lignin content displayed a low but significant negative coefficient for all biomasses (− 0.64), and it was only significant for miscanthus sample (− 0.90) when considering biomasses separately. No correlations were observed for wheat straw alone as the glucose yields were as high for HW- and DA-pretreated samples as for the delignified CHLO-pretreated samples. G unit content coefficient was found to have the most important coefficients when considering all biomasses together (− 0.82). It was also statistically significant for poplar samples alone (− 0.93) and relatively high for wheat straw (− 0.81) and miscanthus samples (− 0.88). S unit content displayed a lower negative coefficient for all biomasses (− 0.54) and was not significant for any biomass considered alone, although the values were relatively high. These results suggest G units are more detrimental to enzymatic hydrolysis as branched G-rich lignin can give rise to more resistant physical barrier around the cellulose fibrils [18]. Although lignin composition displayed high coefficients for all the biomasses, the S/G ratio was not a good indicator of biomass recalcitrance. The only set of samples displaying a high coefficient was the miscanthus samples (− 0.90). However, as the values were very similar for all miscanthus samples, it is likely that this high coefficient is irrelevant. The importance of lignin structure in biomass recalcitrance was outlined by the negative coefficient calculated for the proportion of β-O-4′ linkages for all biomasses (− 0.63). Interestingly, when considering each biomass separately, this factor seemed less important as lignin content coefficient increased. Accordingly a very high coefficient was obtained for wheat straw samples (− 0.99), while no correlation was observed with lignin content. On the contrary, no correlation was observed with the β-O-4′ linkages for the miscanthus samples, while the correlation with the lignin content was statistically significant. A possible explanation might be that lignin conformation would become the main factor affecting enzymes access to the polysaccharides when the amount of lignin is reduced as it would influence the way it fills pores or covers cellulose fibrils. The negative correlation between saccharification yields and both lignin structure and content was strengthened by the strong negative coefficient obtained for fluorescence intensity, either considering all biomasses (− 0.73) or each biomass separately, with statistically significant values obtained for miscanthus (− 0.99) and poplar (− 0.90). Fluorescence was previously found to be a relevant predictive marker of enzymatic digestibility of steam-exploded biomass samples [59]. Although the exact mechanism of plant cell wall fluorescence needs to be further investigated, our results indicate that fluorescence intensity can be considered as a good marker of biomass susceptibility to enzymatic hydrolysis.

#### Structural features

Statistically significant negative correlations were observed between glucose yield and fibrils’ diameter for all biomasses (− 0.57), which is in agreement with the contribution of lignin in the microfibrils’ aggregates [43]. However, this value remained low compared to the lignin-related coefficients discussed previously, considering either the three biomasses together or separately. This can be explained by the interactions of hemicellulose within the lignified amorphous matrix that holds together the cellulose microfibrils.

The correlations between the cell wall’s porosity and the saccharification yields displayed some interesting results. While some high-value coefficients were obtained when considering the biomass samples separately, no correlations were observed for the three biomasses together. For wheat straw, strong positive coefficients (0.7–0.9) were observed for porosity threshold between 15 and 30 nm even if only the overall porosity below 30 nm was statistically significant (*p* = 0.02). Higher coefficient values for lower porosities could have been expected, as some fungal enzymes are known to have small hydrodynamic radii around 2.0–3.5 nm [60, 61]. However, samples did not display an increase in this specific pore size except for the CHLO sample (Fig. 5), which may explain weak correlation value. For the miscanthus samples, only pores below 10 nm showed a strong positive correlation coefficient. High correlations were obtained for the poplar samples, especially when considering the small pores (< 15 nm). Interestingly, wheat straw displayed the highest coefficients, whereas untreated wheat straw had the lowest porosity among the three biomasses. This can be explained by the higher improvement of plant cell wall’s porosity induced by the different pretreatments compared to what was observed for poplar and miscanthus (Fig. 5). The correlations obtained for different pore sizes for each biomass and the absence of correlation when considering all the samples indicate that there is no generic pore size allowing an improvement of saccharification and that enzymes’ diffusion within the plant cell wall is specific to each biomass species. An increase in porosity does not necessarily mean that cellulose surface is made more accessible. This can also lead to an enhancement in the accessibility to the lignin and so to an increase in its unproductive binding to enzymes [62, 63]. The increase in porosity and lignin removal are likely to be intertwined as lignin has a well-known pore-filler role [17].

Our results tend to show that lignin removal has a more important impact on saccharification efficiency than the increase in porosity. Modifying lignin structure seems essential as both size and composition of the polymer can vary, impacting the way lignin covers cellulose fibrils. Decreasing lignin content would then allow increasing porosity and improving enzymes diffusion inside the plant cell walls, but the removal of the physical barrier formed by lignin around cellulose at the molecular scale needs to be efficiently achieved for enzymes to access their substrate. Similar conclusions were drawn in previous studies focusing on more restricted combination of biomasses and pretreatments. Xu et al. used principal component analysis to show that surface area and pore volumes were well correlated with the enzymatic hydrolysis of rice straw pretreated using cholinium ionic liquid and negatively related to lignin content [64]. Increasing cellulose accessibility was found to be more important than reaching a low lignin content to enhance the saccharification of SO_2_^−^ catalysed steam-exploded spruce samples [65]. The major importance of increasing cellulose accessible area by removing the surrounding lignin to enhance saccharification efficiency was also demonstrated on pretreated switchgrass [66] and wheat straw [67]. These concurring conclusions show that lignin plays an important role in governing cellulose accessibility by its presence in the plant cell wall and probably more importantly by its structure and organisation around the cellulose fibrils.

## Conclusions

The multimodal analysis of biomass samples of different botanical origins submitted to contrasted pretreatments allowed determining correlations between the samples’ structural, chemical and spectral features on the one hand and their final saccharification yields on the other hand. Our results highlight some generic markers of plant cell wall recalcitrance. Regarding structural markers, porosity was found to be a critical feature, but the pore size thresholds that correlate the best vary depending on the biomass species. Saccharification could then be improved by engineering enzymatic cocktails depending on the biomass feedstock in order to adapt enzymes’ size to the porosity of the considered biomass. More information on porosity’s impact on enzymes' activity could be obtained by analysing the modification of cellulose accessible surface, or by observing the distribution and diffusion of enzymes within the plant cell wall using confocal microscopy techniques [68]. The correlations obtained for structural features were, however, less strong than some obtained for chemical features. Among them, lignin composition (in particular S and G units content and aryl ether linkages content) seems at least as important as lignin content to explain biomass recalcitrance. This probably reveals that nano-chemical structure and composition of the cell walls, which was not investigated, depends on biomass species, and is likely directly responsible for the different saccharification potential. Further insights in the molecular weight and organisation of the residual lignin could provide a better understanding on lignin reorganisation during the pretreatments. Fluorescence intensity was found to be a good general predicting factor of the saccharification potential and may allow rapid assessment of the recalcitrance of lignocellulosic biomass. Thus, a deeper understanding of the molecular patterns responsible for fluorescence emission might help improving our knowledge on how lignin structure influences enzymatic hydrolysis.

## Additional file

**Additional file 1: Figure S1.** Morphology of the wheat straw, miscanthus and poplar fragments before and after pretreatments. Scale bars: 5 mm. **Figure S2.** SEM images of the transverse surface of the different fragments. Samples were imaged with an inclination angle of 45°. Scale bars: 100 μm.

## Abbreviations

2D-HSQC NMR: two-dimensional heteronuclear single-quantum coherence solution nuclear magnetic resonance spectroscopy
CHLO: sodium chlorite
DA: dilute acid
FE-SEM: field-emission scanning electron microscopy
HPLC: high-performance liquid chromatography
HPAEC-PAD: high-performance anion-exchange chromatography with pulsed amperometric detection
HW: hot water
IL: ionic liquid
SD: standard deviation
SEM: scanning electron microscopy
