# Supplementary material for: Multimodal analysis of pretreated biomass species highlights generic markers of lignocellulose recalcitrance
Source: Biotechnol Biofuels. 2018 Feb 27;11:52. doi: 10.1186/s13068-018-1053-8 (PMC5828075; doi:10.1186/s13068-018-1053-8)
Supplement: Supplementary file 1 — Additional file 1: Figure S1. Morphology of the wheat straw, miscanthus and poplar fragments before and after pretreatments. Scale bars: 5 mm. Figure S2. SEM images of the transverse surface of the different fragments. Samples were imaged with an inclination angle of 45°. Scale bars: 100 μm. [file 13068_2018_1053_MOESM1_ESM.docx]

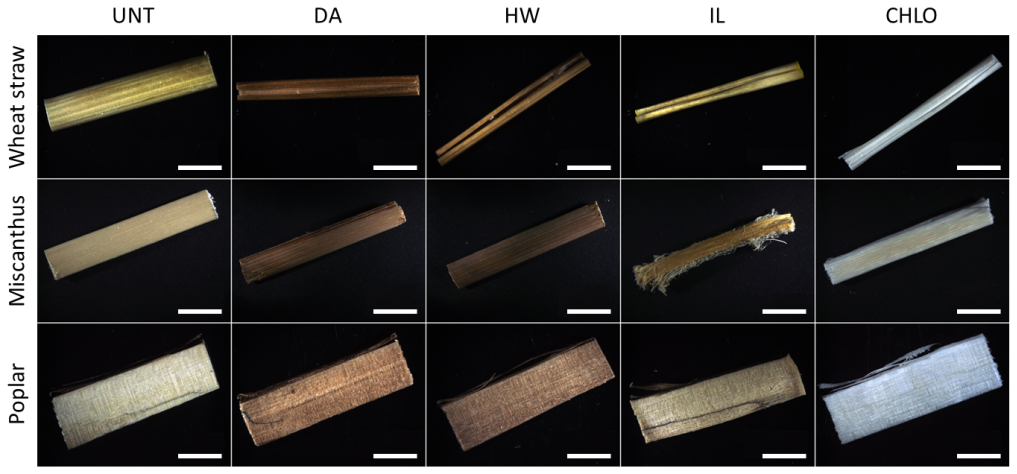


Figure S1. Morphology of the wheat straw, miscanthus and poplar fragments before and after pretreatments. Scale bars: 5 mm.


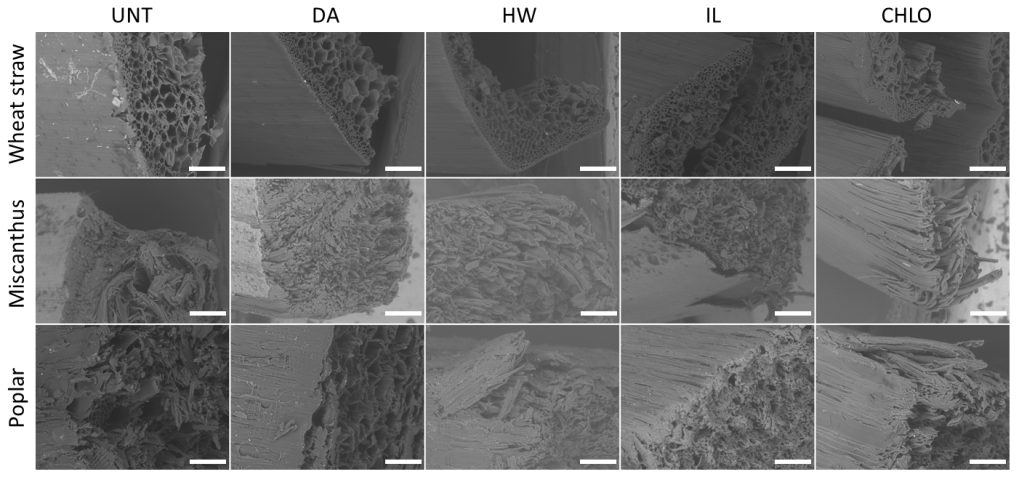


Figure S2. SEM images of the transverse surface of the different fragments. Samples were imaged with an inclination angle of 45°. Scale bars: 100 µm.
